# Supplementary material for: Histone Deacetylases (HDACs): Evolution, Specificity, Role in Transcriptional Complexes, and Pharmacological Actionability
Source: Genes (Basel). 2020 May 15;11(5):556. doi: 10.3390/genes11050556 (PMC7288346; doi:10.3390/genes11050556)
Supplement: Supplementary file 1 [file genes-11-00556-s001.zip › suppFileS1.docx]

**Supplementary File S1**

Histone Deacetylases (HDACs): evolution, specificity, role in transcriptional complexes and pharmacological actionability

By Milazzo, Mercatelli, Di Muzio, Triboli, De Rosa, Perini and Giorgi

The following panel (Supplementary Figure S1) indicates sequence-based classification of HDACs described in this review (also described in main Table 1), defining color schemes used throughout the main manuscript.

**
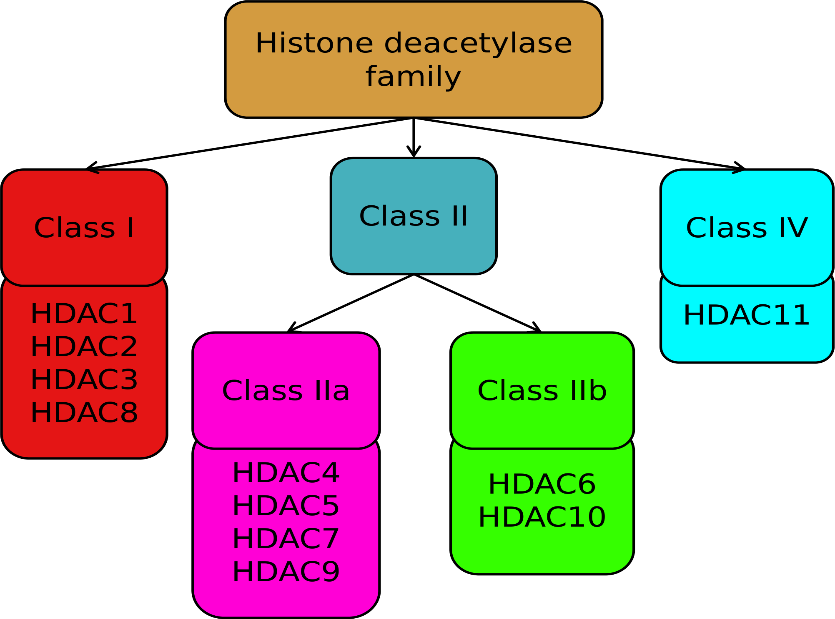
**

**Supplementary Figure S1**. Color codes used in the HDAC classification of this manuscript.

**Domain Analysis**

To perform the domain analysis, we gathered the 11 sequences from Homo sapiens, uploaded on the SMART (PMID 29040681) online tool and retrieved the catalytic domains of each HDAC. For the additional domains, we retrieve them from UniProt. To build Figure 2 A we use the software IBS (Illustrator for Biological Sequences, PMID 26069263). To derive information about the interaction domains in the complexes that HDACs form, we performed a literature search.

**Alternative Splicing**

The number of isoforms generated by alternative splicing varies amongst HDACs of the same class. While some HDACs (1, 2, 3, 4 and 11) have only 1 or 2 alternative isoforms, which is below the average transcript diversity (5.4 isoforms/gene) found for human genes (PMID 17571346), HDAC7 and 9 have 10 and 11 isoforms, respectively. The number of HDAC isoforms reported below refers to *Homo sapiens* (source: Uniprot PMID: 30395287):

HDAC1: 1

HDAC2: 2

HDAC3: 2

HDAC4: 2

HDAC5: 3

HDAC6: 2

HDAC7: 10

HDAC8: 6

HDAC9: 11

HDAC10: 4

HDAC11: 2

**Drosophila/Human Comparative Genomic Analysis**

Unsurprisingly, the chromosomal location of HDACs, unlike protein sequences, is not conserved between vertebrates and invertebrates. We show this as a comparative genome analysis of HDAC locations in Drosophila and human genomes in the following Figure, where we label the location of homologous HDAC genes in Drosophila and Human genomes. In the inner circle, we show the karyotype of both human and Drosophila. Chromosome banding was obtained from the UCSC Genome Browser (PMID 12045153). The location of the different genes has been retrieved from the UCSC Genome Browser (PMID 12045153). We used the dm3 and the hg38 version of the genomes. The Drosophila genome coordinates were multiplied by a factor of 8.75 for visualization purposes. The circular visualization has been built using the R package *circlize* (PMID 24930139).


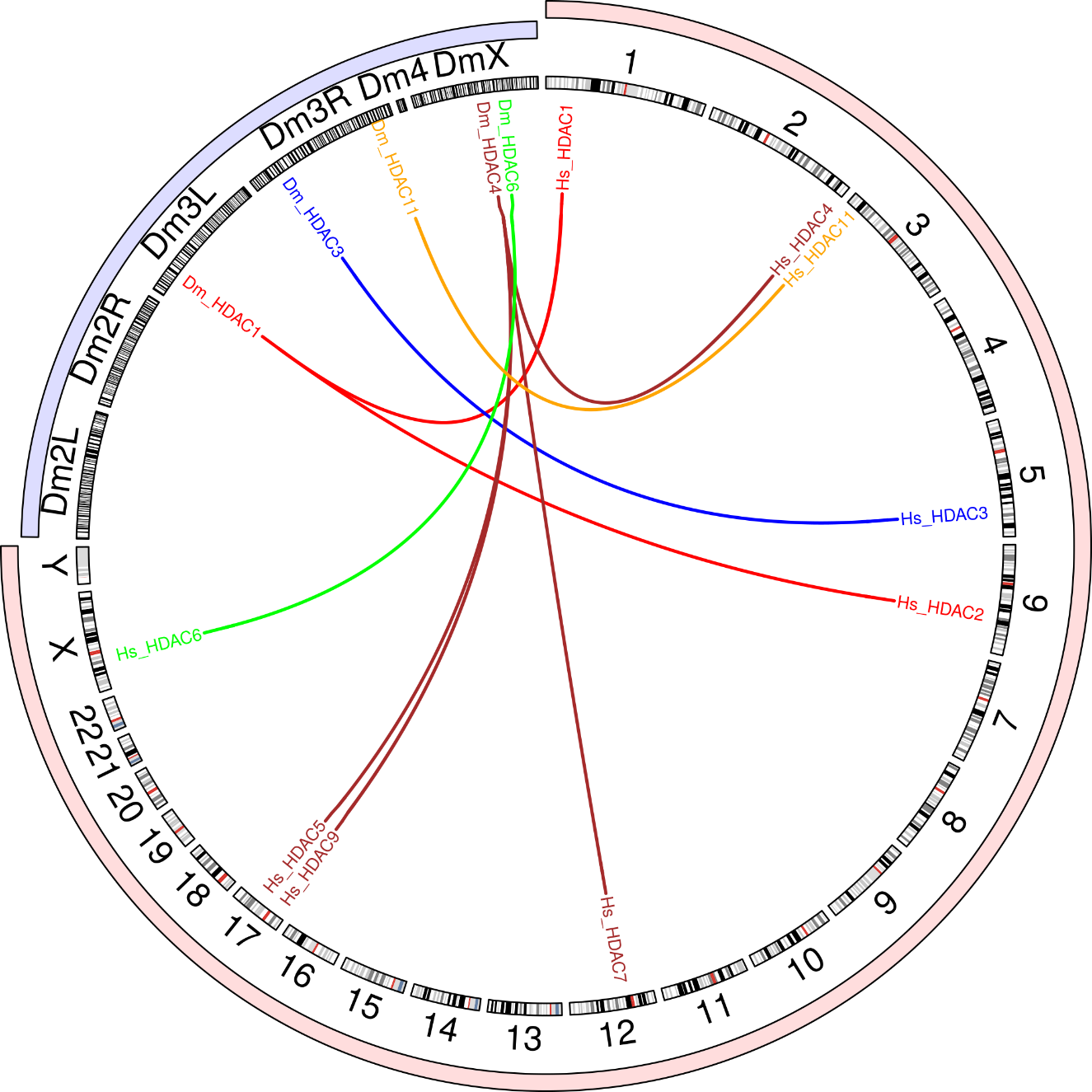


**Supplementary Figure S2**. Circular representation of human (in pink) and Drosophila (in blue) genomes and the chromosomal location of HDAC orthologous pairs.

**HDACs Gene Expression Profile in Normal and Tumor Tissues**

The transcriptional profiles of genes encoding HDAC1-11 were generated using normal tissues RNA-seq datasets available from the GTEx Consortium [PMID: 23715323] and tumor tissues RNA-seq datasets available from the TCGA database [PMID: 29625045]. Neuroblastoma RNA-seq datasets were download from the Therapeutically Applicable Research to Generate Effective Treatments (TARGET)-NBL database (https://ocg.cancer.gov/programs/target/data-matrix). The FPKM normalization (Fragments Per Kilobase of transcripts per Million mapped reads) was used to show the expression level of transcripts. We noticed a discrepancy with regards to HDAC10 compared to expression levels expressed in TPMs showed at the GTEx online portal (accessed on March 27th 2020). FPKM normalization was performed using the USCS data on the longest transcript isoform. The isoform used for normalization was ENST00000216271.10, 2567 nts long, which provided us expression values slightly lower (even if correlated to) those available online at the GTEx Portal. We acknowledge the TCGA Research Network, and the GTEx Consortium Portal, for providing the data required for these analyses.

**Survival analysis**

The TCGA and the TARGET clinical data were downloaded from the respective data portals. Kaplan–Meier curves were used to identify the relationship between HDACs gene expression level and the overall survival in different tumors by stratifying the patients two groups (high and low expression), considering the median expression value for a given HDAC as the threshold for group assignment. Significancy of the analysis was screened via the survival R package. The p-value was obtained using the log-rank test and a p < 0.05 were considered as statistically significant.
